# Real-world uptake of *gBRCA* testing as a companion diagnostic for olaparib in patients with high-risk HER2-negative early breast cancer in Japan: a cross-sectional multicenter study (BRCAwareness)

**DOI:** 10.1007/s12282-026-01867-y

**Published:** 2026-05-23

**Authors:** Tomoe Taji, Yukari Uemura, Yuri Kimura, Noriko Maeda, Aki Ito, Hirohito Seki, Daisuke Takabatake, Michiko Harao, Shogo Nakamoto, Ryoichi Matsunuma, Chinatsu Koganezawa, Takayuki Iwamoto, Hirofumi Mukai, Yuichiro Kikawa

**Affiliations:** 1 Department of Breast Surgery, Kansai Medical University, 3-1 Shinmachi 2 Chome, Hirakata, Osaka 573-1191 Japan; 2 Laboratory of Biostatistics, Department of Data Science, Center for Clinical Sciences, Japan Institute for Health Security, 1-21-1 Toyama, Shinjuku-ku, Tokyo, 162-8655 Japan; 3 Department of Breast Surgical Oncology, The Cancer Institute Hospital of Japanese Foundation for Cancer Research, 3-8-31, Ariake, Koto-ku, Tokyo, 135-8550 Japan; 4 Department of Gastroenterological, Breast and Endocrine Surgery, Yamaguchi University Graduate School of Medicine, 1-1-1, Minami Kogushi, , Ube, Yamaguchi 755-8505 Japan; 5 Department of Breast Surgery, Akita Red Cross Hospital, 222-1 Kamikitatesarutanawashirosawa, Akita, Akita 010-1495 Japan; 6 Department of Breast Surgery, National Hospital Organization Tokyo Medical Center, 2-5-1 Higashigaoka Meguro-ku, Tokyo, 152-8902 Japan; 7 Department of Breast Surgery, National Hospital Organization Shikoku Cancer Center, 160 Minamiumemotomachi, Matsuyama, Ehime 791- 0245 Japan; 8 Department of Breast Oncology, Jichi Medical University, 3311-1 Yakushiji, , Shimotsuke, Tochigi 329-0498 Japan; 9 Department of Breast and Endocrine Surgery, Okayama University Hospital, 2-5-1 Shikata-cho, Kitaku, Okayama 700-8558 Japan; 10 Department of Breast Surgery, Shizuoka General Hospital, 4-27-1 Kita-ando, Aoi-ku, Shizuoka, 420-8527 Japan; 11 Department of Breast Oncology, Hokkaido Cancer Center, 2-3-54 Kikusui 4-jo, Shiroishi-ku, Sapporo, Hokkaido 003-0804 Japan; 12 Department of Breast and Thyroid Surgery, Kawasaki Medical School, 577 Matsushima, Kurashiki, Okayama 701-0192 Japan; 13 Department of Medical Oncology, National Cancer Center Hospital East, 6-5-1 Kashiwanoha, Kashiwa, Chiba 277-8577 Japan

**Keywords:** *BRCA*, Olaparib, High-risk early breast cancer

## Abstract

**Background:**

Adjuvant olaparib significantly improves invasive disease-free and overall survival in high-risk human epidermal growth factor receptor 2 (HER2)-negative, early breast cancer patients carrying germline breast cancer susceptibility gene 1/2 (*gBRCA*) pathogenic variants (PVs). Timely *gBRCA* testing as a companion diagnostic for adjuvant olaparib is essential. However, its real-world uptake remains unclear.

**Methods:**

We enrolled patients with invasive HER2-negative early breast cancer who underwent curative surgery during 2023 in Japan. Eligibility was based on the OlympiA trial criteria. Estrogen receptor (ER)-positive patients required ≥ 4 positive nodes after surgery or non-pathological complete response (non-pCR) with clinical and pathologic stage (CPS) and estrogen receptor status and histologic grade (EG) score ≥ 3 following neoadjuvant chemotherapy (NAC). ER-negative patients required invasive tumor > 2 cm or ≥ 1 nodal metastasis after surgery, or non-pCR after NAC. The primary outcome was *gBRCA* testing rate; secondary outcomes included a proportion of patients with *gBRCA* PVs and a proportion of patients starting adjuvant olaparib. We also explored factors associated with not undergoing testing.

**Results:**

Of 824 patients enrolled from 46 facilities, 691 were analyzed after random sampling and exclusions. The testing rate was 63.2% (95% confidence interval 59.5–66.9). Among 254 untested patients, 168 (66%) were not informed—mainly due to physician oversight in recognizing eligibility (57%) or physician-perceived patient ineligibility (40%). Of 69 informed but untested patients, reasons included psychological distress (46%), testing cost (35%), and familial concerns (12%). Of 42 patients (9.6%) with *gBRCA* PVs, 32 received olaparib. Multivariable analysis (female only) showed that age ≥ 65 years, postmenopausal status, major comorbidities, upfront surgery, absence of family history of hereditary breast and ovarian cancer-related cancers, and absence of bilateral or multiple primary breast cancers were associated with lower testing rates.

**Conclusion:**

Greater physician awareness of companion diagnostic is needed to ensure timely *gBRCA* testing and equitable access to adjuvant olaparib.

**Supplementary Information:**

The online version contains supplementary material available at 10.1007/s12282-026-01867-y.

## Introduction

The number of breast cancer patients in Japan has been gradually increasing, with 101,793 new cases diagnosed among Japanese women in 2022 [1]. Hereditary breast and ovarian cancer (HBOC) is the most common cancer predisposition syndrome associated with breast cancer, caused by pathogenic variants (PVs) in germline *BRCA1 /2 (gBRCA)* [2]. A diagnosis of HBOC enables the implementation of risk-reducing strategies, including intensified surveillance, chemoprevention, and risk-reducing surgery.

Poly (ADP-ribose) polymerase (PARP) inhibitors have demonstrated clinical benefit in patients with breast cancer carrying *gBRCA* PVs. In particular, the phase III OlympiA trial showed that adjuvant olaparib significantly improved invasive disease-free survival (IDFS) and overall survival (OS) in high-risk human epidermal growth factor receptor 2 (HER2)-negative early breast cancer patients with g*BRCA* PVs [3,4]. Recent real-world analyses have attempted to estimate the proportion of patients who meet the high-risk criteria defined in the OlympiA trial. Population-based and institutional studies from Europe have reported that approximately 13–14% of patients with HER2-negative early breast cancer meet the OlympiA high-risk criteria, suggesting that a considerable number of patients may require *gBRCA* testing to determine eligibility for adjuvant olaparib [5,6].

In Japan, national health insurance covers *gBRCA* testing for patients with breast cancer in three clinical settings: (1) as a companion diagnostic for PARP inhibitors in patients with HER2-negative metastatic or recurrent breast cancer since 2018; (2) for the diagnosis of HBOC since 2020; and (3) as a companion diagnostic for adjuvant olaparib in patients with high-risk HER2-negative early breast cancer since 2022.

Although genetic testing is essential to determine eligibility for adjuvant olaparib, the real-world testing rate in Japan remains unclear. The lack of studies reporting testing rate may be due to the complex eligibility criteria used in the clinical trial [3,4] and the relatively short time since national insurance coverage began. Patients who are not tested may miss the opportunity to receive adjuvant olaparib, which could adversely affect their outcome.

Identifying barriers to testing and implementing strategies to improve uptake may help ensure access to adjuvant olaparib among patients with high-risk HER2-negative early breast cancer and *gBRCA* PVs. Therefore, we conducted a multicenter cross-sectional study to evaluate the proportion of patients with high-risk HER2-negative early breast cancer who underwent g*BRCA* testing and understand reasons for testing or not seeking testing.

## Patients and methods

### Patients

This study is reported in accordance with the STROBE statement (Table S1). We conducted a cross-sectional study using routinely collected medical records. Patients in Japan with newly diagnosed invasive HER2-negative early breast cancer who underwent curative surgery between January 1 and December 31, 2023 were included. Curative surgery was defined as the index surgery.

The most complex eligibility criterion for postoperative olaparib applies to patients with estrogen receptor (ER)-positive breast cancer who received neoadjuvant chemotherapy (NAC), requiring both non–pathological complete response (non-pCR) and a clinical and pathologic stage plus estrogen receptor status and histologic grade (CPS + EG) score of ≥ 3 (Table S2). Therefore, all non-pCR cases were included in the primary registration. The CPS + EG score was centrally calculated to determine eligibility, and patients eligible (CPS + EG score ≥ 3) for secondary registration were selected accordingly. For primary registration, the inclusion criteria were no distant metastasis, age ≥ 18 years at diagnosis, and high risk of recurrence. High risk was defined as follows. For ER-positive patients: (1) four or more lymph node metastases (pN2 or higher) after upfront surgery, or (2) non-pCR following NAC. For ER-negative patients: (1) an invasive tumor size > 2 cm (pT2 or higher) or at least one lymph node metastasis (pN1 or higher) after upfront surgery, or (2) non-pCR following NAC. Prior to primary registration, the principal investigator at each institution was requested to consecutively identify all eligible patients from surgical cases in 2023.

### Data collection

Patient-level data were collected from medical records, whereas facility-level data were obtained from the principal investigator at each participating institution. These data included presence of certified genetic counselors, geneticists, certified nurses, the number of breast specialists, annual breast cancer surgery cases, patients’ age, sex, menopausal status, family history of cancer, past medical history, comorbidities, clinical and pathological stage, information about pathology, surgery, drug therapy, and *gBRCA* testing. Information on whether testing was explained or offered was primarily obtained from chart review at each institution. If no documentation was found, the treating physician was contacted to confirm. If this information remained unavailable, the case was classified as “unknown.” Patients were considered to have undergone *gBRCA* testing if the test was performed before the index surgery or within 365 days after the index surgery. Tests performed after the diagnosis of metastatic or recurrent disease were excluded.

### Outcome measures

The primary outcome was the proportion of patients who underwent *gBRCA* testing, defined as the number of patients who received *gBRCA* testing divided by the number of patients who met the secondary registration eligibility criteria (CPS + EG score ≥ 3). Secondary outcomes were a proportion of patients with g*BRCA* PVs among tested patients, a proportion of patients initiated on olaparib among g*BRCA* PV holders, and systemic therapies used other than olaparib in g*BRCA* PV carriers. We also explored factors associated with not undergoing *gBRCA* testing.

### Statistical analysis

We set the expected proportion at 88% as a primary outcome. This was because 87% of young women with breast cancer received *gBRCA1/2* testing in the United States [7], and we anticipate a higher proportion to avoid missing eligible patients for adjuvant olaparib.

With an 88% expected proportion of tests, a total of 688 cases is needed to maintain a 95% confidence interval (CI) width of 0.05. The final sample size, accounting for potential exclusions, is set at 700. Additionally, sample sizes for other scenarios, including alternative estimated proportions, were also assessed (Table S3).

After primary registration, among ER-positive patients with non-pCR after NAC, those with a CPS + EG score of less than 3 points were excluded. We then performed random sampling to select 700 eligible cases for secondary registration. To ensure representation across sites, we aimed to include at least one patient from each institution. Exceptions were permitted when an institution had no eligible patients or when enforcing this rule would cause substantial selection bias.

For the analysis set, we estimated the proportion of patients who underwent *gBRCA* testing and calculated 95% confidence intervals (CIs). To identify factors associated with not undergoing *gBRCA* testing (outcome: not tested), we fitted logistic regression models and reported odds ratios (ORs) with 95% CIs. Multivariable models included pre-specified baseline factors selected a priori based on clinical relevance: age, sex, parity (women only), severe comorbidities, family history of HBOC-related cancers, two or more primary breast cancers, bilateral breast cancer, tumor subtype (luminal vs. triple-negative), Japanese Organization of Hereditary Breast and Ovarian Cancer (JOHBOC)-designated facility certification, availability of genetic specialists, availability of certified nurses, number of breast specialists, presence of perioperative conferences, and annual breast cancer surgery volume.

Statistical analysis was performed using SAS version 9.4. A two-sided test was adopted, with *p* < 0.05 indicating statistical significance.

### Ethical considerations

The study protocol was approved by the Ethics Committee of Kansai Medical University Hospital (approval number 2024204). The need for patient consent was waived because the patient records were anonymized and de-identified before analysis.

## Results

**Fig. 1:**
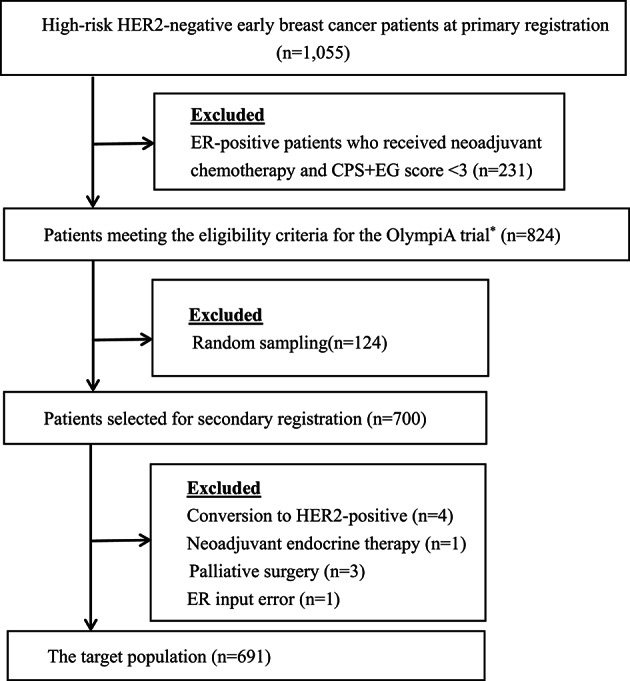
Patient Flow Diagram. *HER2* human epidermal growth factor receptor 2, *ER* estrogen receptor, *CPS* clinical and pathologic stage, *EG* estrogen receptor status and histologic grade*Geyer CE et al. Ann Oncol. 2022;33(12):1250–1268

For primary registration 1,055 patients were enrolled from 46 facilities. After excluding 231 patients with a CPS + EG score < 3,824 patients met the inclusion criteria for adjuvant olaparib. Of these, 700 patients were selected for secondary registration by random sampling. We confirmed that there were no numerically significant differences in background factors (age, sex, subtype, and treatment plan) between the primary cohort of 824 enrolled patients and the secondary cohort of 700 selected patients (Table S4). Among the 700 patients, 691 patients were analyzed after excluding 9 ineligible patients. Of the nine ineligible cases, four had HER2-positive disease, one was excluded due to a data entry error, and one received neoadjuvant endocrine therapy. The remaining three patients deviated from standard treatment: two initially refused surgery and received capecitabine until disease progression before undergoing surgery, whereas one refused surgery after NAC and underwent surgery after disease progression (Fig. 1).

The median age was 62 (range, 20–96) years, 99% were female, 25 (3.6%) had two or more primary breast cancers, 52 (7.5%) had bilateral breast cancer, 347 (50.2%) were ER-positive and NAC was administered to 362 patients (52.4%) (Table 1). Regarding facility characteristics, the median number of breast cancer specialists per facility was 3 (range, 1–17), and genetic counselors were available in 27 facilities (58.7%) (Table 2).

**Table 1:** Demographics and other baseline characteristics

		*N* = 691
Age (year)		62 [20–96]
Sex	Female	684 (99.0%)
Neoadjuvant chemotherapy	Yes	362 (52.4%)
Subtype	Triple negative	344 (49.8%)
	Luminal	347 (50.2%)
ER	≥ 10%	343 (49.6%)
	1–9%	12 (1.7%)
	0%	336 (48.6%)
Menopausal status*^1^	Premenopausal	203 (29.7%)
	Postmenopausal	479 (70.0%)
	N/A	2 (0.3%)
Parity*^1^	No	158 (23.1%)
	Yes	513 (75.0%)
	N/A	13 (1.9%)
Family history	Breast cancer	157 (22.7%)
	Ovarian cancer	33 (4.8%)
	Pancreatic cancer	43 (6.2%)
Two or more primary breast cancers	Yes	25 (3.6%)
Bilateral breast cancer	Yes	52 (7.5%)
Comorbidities	Malignancies other than BC	50 (7.2%)
	Dementia	18 (2.6%)
	Other severe comorbidities	22 (3.2%)
Breast surgery	Total mastectomy	541 (78.3%)
	Partial mastectomy	149 (21.6%)
	N/A	1 (0.1%)
Risk-reducing surgery	RRM	6 (0.9%)
	RRSO	10 (1.4%)
	RRM+RRSO	4 (0.6%)
Neoadjuvant chemotherapy*^2^	ICIs	81 (22.4%)
	Without ICIs	281 (77.6%)
Adjuvant chemotherapy	No	265 (38.4%)
	ICIs	47 (6.8%)
	Without ICIs	379 (54.8%)
Adjuvant olaparib	Yes	32 (4.6%)
Adjuvant abemaciclib	Yes	237 (34.3%)

*N* (%), median [range]

*RRM* risk-reducing mastectomy, *RRSO* risk-reducing salpingo-oophorectomy, *ICIs* immune checkpoint inhibitors

*^1^ Denominator = number of female patients

*^2^ Denominator = number of patients receiving neoadjuvant chemotherapy

**Table 2:** Information of the facilities

		*N* = 46
Certified genetic counselor	Yes	27 (58.7%)
	Full-time	24 (52,2%)
	Part-time	3 (6.5%)
Certified clinical geneticist^*1^	Yes	36 (78.3%)
Number of specialists^*2^		3 [1–17]
Certified nurse^*3^	Yes	42 (91.3%)
Number of breast cancer surgeries in 2023	< 50	1 (2.2%)
	50–99	6 (13.0%)
	100–199	20 (43.5%)
	200–299	10 (21.7%)
	300–399	6 (13.0%)
	400 ≤	3 (6.5%)
Accreditation of facilities from JOHBOC	Yes	25 (54.3%)
Pick up HBOC suspicious cases using a medical questionnaire	Yes	29 (63.0%)
Perioperative conference	Yes	42 (91.3%)

N (%), median [range]

*JOHBOC* Japanese Organization of Hereditary Breast and Ovarian Cancer; *PV*, pathogenic variant; *VUS*, variant of uncertain significance

*1 Certified Japanese Board of Medical Genetics and Genomics, Clinical Genetics or Clinical Genetics or Board-certified Doctor Member of the Japanese Society for Hereditary Tumors

*2 Breast Cancer Specialist of The Japanese Breast Cancer Society

*3 Certified Nurse in Breast Cancer Nursing or Certified Nurse Specialist in Cancer Nursing

**Fig. 2:**
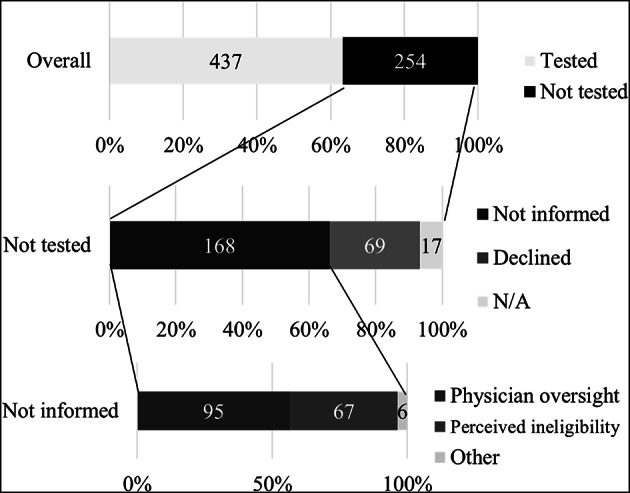
*gBRCA* testing status and reasons for non-testing. N/A, not available

As a primary outcome, *gBRCA* testing was performed in 437 of 691 patients (63.2% [95% CI 59.5–66.9]). Substantial inter-institutional variability in *gBRCA* testing rates was observed (Figure S1). Of 254 untested patients, 168 (66.1%) had no documented physician explanation or offer of testing, mainly due to physician oversight in recognizing eligibility (56.5%) or physician-perceived patient ineligibility related to age, comorbidities, or treatment refusal (39.9%). Among 69 patients who were informed but declined testing, the reasons for not testing were psychological distress (46.4%), cost of testing (34.8%), and concerns about genetic implications for family members (11.6%) (Fig. 2, S1). A total of 42 patients (9.6%) were carriers of *gBRCA* PVs, of whom 27 had *gBRCA1*, 14 had *gBRCA2*, and 1 carried PVs in both *gBRCA1* and *gBRCA2* (Table 3, Figure S1). By subtype, *gBRCA* PVs were detected in 32 of 220 patients with triple-negative breast cancer (14.5%) and in 10 of 217 patients with luminal tumors (4.6%) (Table S5). Among 42 patients with g*BRCA* PVs, 32 received adjuvant olaparib. Of the remaining 10 patients, all but three underwent perioperative chemotherapy, and none received abemaciclib (Figure S1).

**Table 3:** Results of g*BRCA* testing

		*gBRCA1*			Total
		PV (+)	VUS	PV (-)	
*gBRCA2*	PV (+)	1	0	14	15
	VUS	2	0	9	11
	PV (-)	25	2	384	411
Total		28	2	407	437

**Fig. 3:**
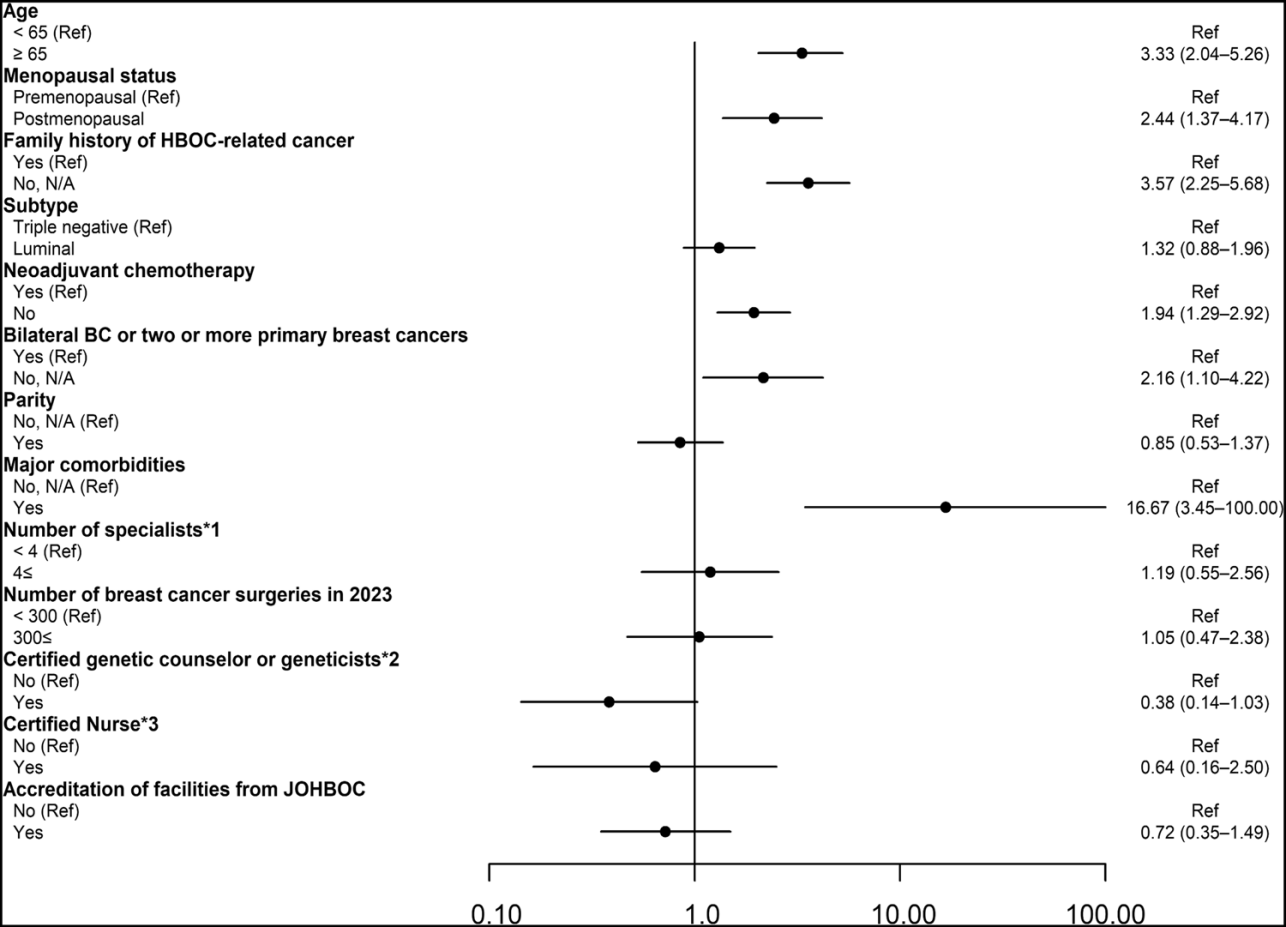
Multivariable analysis of factors associated with not being tested. *FH* family history, *HBOC* hereditary breast and ovarian cancer, *BC* breast cancer, *CGC* certified genetic counselor, *JOHBOC* Japanese Organization of Hereditary Breast and Ovarian Cancer

The testing rate was far lower than 88%. We performed a predefined multivariable analysis (Table 4; Fig. 3). Age ≥ 65 years (OR 3.33, 95% CI 2.04–5.26), postmenopausal status (OR 2.44, 95% CI 1.37–4.17), major comorbidities (OR 16.67, 95% CI 3.45–100.0), upfront surgery (OR 1.94, 95% CI 1.29–2.92), absence of family history of HBOC-related cancer (OR 3.57, 95% CI 2.25–5.68), and absence of bilateral breast cancer or multiple primary breast cancers (OR 2.16, 95% CI 1.10–4.22) were associated with non-testing.

**Table 4:** Multivariable analysis of factors associated with not undergoing *gBRCA* testing (women only)

		Tested	Not tested	Odds ratio	95% CI		*P* value
		*N* = 431	*N* = 253				
**Age**	< 65	302	81	ref			
	≥ 65	129	172	**3.33**	**2.04**	**5.26**	**< 0.0001**
**Menopausal status**	Premenopausal	174	31	ref			
	Postmenopausal	257	222	**2.44**	**1.37**	**4.17**	**0.0024**
**Family history of HBOC-related cancer**	Yes	165	40	ref			
	No, N/A	266	213	**3.57**	**2.25**	**5.68**	**< 0.0001**
Subtype	Triple negative	219	124	ref			
	Luminal	212	129	1.32	0.88	1.96	0.1753
**Neoadjuvant chemotherapy**	Yes	263	96	ref			
	No	168	157	**1.94**	**1.29**	**2.92**	**0.0016**
**Bilateral BC or multiple primary BCs**	Yes	50	19	ref			
	No, N/A	381	234	**2.16**	**1.10**	**4.22**	**0.0245**
Parity	No, N/A	112	59	ref			
	Yes	319	194	0.85	0.53	1.37	0.4975
**Major comorbidities**	No, N/A	429	217	ref			
	Yes	2	36	**16.67**	**3.45**	**100.0**	**0.0004**
Number of specialists^*1^	< 4	225	145	ref			
	4 ≤	206	108	1.19	0.55	2.56	0.6558
Number of breast cancer surgeries in 2023	< 300	269	185	ref			
	300 ≤	162	68	1.05	0.47	2.38	0.8972
Certified genetic counselor or geneticists^*2^	No	31	34	ref			
	Yes	400	219	0.38	0.14	1.03	0.0563
Certified Nurse^*3^	No	14	14	ref			
	Yes	417	239	0.64	0.16	2.50	0.5198
Accreditation of facilities from JOHBOC	No	131	108	ref			
	Yes	300	145	0.72	0.35	1.49	0.3739

Bold values indicate statistically significant factors and their corresponding ORs, 95% CIs, and P values

*JOHBOC* Japanese Organization of Hereditary Breast and Ovarian Cancer; *BC* breast cancer; N/A not available

*1 Breast Cancer Specialist of the Japanese Breast Cancer Society, *2 Certified Genetic Counselor or Certified Japanese Board of Medical Genetics and Genomics, Clinical Genetics, *3 Certified Nurse in Breast Cancer Nursing or Certified Nurse Specialist in Cancer Nursing

## Discussion

This multicenter cross-sectional study is the first large-scale investigation to evaluate the real-world uptake of *gBRCA* testing as a companion diagnostic for adjuvant olaparib in Japan. The overall testing rate was 63.2% (95% CI, 59.5–66.9), which was lower than the anticipated rate of 88%, and as a result, the width of the confidence interval became wider than 0.05, the prespecified CI width.

The main reason for not undergoing testing was that patients had not been informed about the test by their physicians. Among those who declined testing, major barriers included cost and psychological distress related to the test results. These findings are consistent with those of a U.S. online survey, which identified physician recommendation as an important facilitator of testing, while cost and anxiety about test results were reported as major barriers [8].

Previous studies have reported widely varying rates of *gBRCA* testing, reflecting differences in patient populations and study settings. For example, testing was performed in only 31% of patients with HER2-negative metastatic breast cancer in an analysis of the US Flatiron Health database (2014–2022) [9]. In contrast, a Japanese multicenter retrospective study reported a rate of 55.4% among non-metastatic patients undergoing evaluation for HBOC (2020–2021) [10], whereas a US study of young patients with breast cancer (≤ 40 years) reported a testing rate of 87% [7]. However, none of these studies specifically investigated *gBRCA* testing uptake as a companion diagnostic for adjuvant olaparib, which was the focus of the current study. Companion diagnostics are clinically important, as they directly influence treatment decisions and can impact patient outcomes. Therefore, we assumed a testing rate slightly higher than the 87% reported by Rosenberg et al. and set it 88%. Nevertheless, the actual uptake observed in our study was 63.2%—higher than the reported 55.4% in Japan, but still suboptimal—highlighting the need for further improvement in clinical practice. This suboptimal overall uptake may reflect lower testing rates in groups other than very young patients. Among women aged 40 years and younger at breast cancer diagnosis, the testing rate was found to be 98% (49/50), higher than 87%.

Several factors appear to have contributed to the low uptake of *gBRCA* testing. First, a limited time has passed since the indication of *gBRCA* testing was expanded to high-risk HER2-negative breast cancer in 2022. Second, some patients or their physician may think it was unlikely they had *gBRCA* PVs. Third, anticipated psychological distress associated with receiving positive test results, particularly concerns regarding prognosis and potential implications for family members, may have discouraged some patients from undergoing testing or led them to prioritize immediate treatment-related decisions over genetic evaluation. All of these factors have also been identified as reasons for non-testing in the previous study [7]. Fourth, the complexity of clinical trial eligibility criteria may lead to physician oversight. Among the 95 overlooked patients, smaller proportion had characteristics suggestive of HBOC: none were male (1% in the overall cohort), 5.3% were aged ≤ 45 years (14.9% overall), 1.1% had bilateral breast cancer (7.5% overall), and 8.4% had a family history of HBOC-related cancers (29.8% overall). While *gBRCA* testing for HBOC diagnosis can be considered at the time of initial diagnosis, *gBRCA* testing performed as a companion diagnostic for postoperative olaparib often requires postoperative pathological assessment, particularly confirmation of non–pCR in patients treated with NAC. Accordingly, in the present study, more than 40% of patients underwent *gBRCA* testing after surgery (Table S6). Fifth, physicians may have considered older patients (≥ 65 years) or those not receiving perioperative chemotherapy ineligible for adjuvant olaparib, and consequently did not pursue *gBRCA* testing, given that such populations were largely unrepresented in the OlympiA trial. Finally, substantial inter-institutional variability in testing rates was observed (Figure S1). No apparent geographic pattern was identified at the prefecture level (Figure S2), and none of the measured institution-level variables were significantly associated with testing uptake in the multivariable analysis. These findings suggest that the observed differences may be driven by unmeasured factors, such as differences in clinical workflows, referral pathways, or physician awareness, rather than by structural characteristics alone.

Multivariable analysis identified age ≥ 65 years, postmenopausal status, major comorbidities, upfront surgery, absence of family history of HBOC-related cancers, and absence of bilateral or multiple primary breast cancers were associated with lower testing rates (Table 4; Fig. 3). These characteristics may lead clinicians to underestimate the likelihood of *gBRCA* PVs, which could inadvertently result in missed opportunities for targeted therapy. Although not statistically significant, facilities without genetic specialists tended to have a higher proportion of untested patient. Given that 9.6% of tested patients carried PVs and that most received adjuvant olaparib, improving testing coverage is essential to ensure equitable access to effective treatment.

In the present cohort, the prevalence of *gBRCA1* and *gBRCA2* PVs was 6.4% (28/437) and 3.4% (15/437), respectively, which was higher than that reported in an unselected Japanese breast cancer population (1.75% and 2.71%) [2]. In contrast, the higher frequency of *gBRCA* PVs in triple-negative breast cancer (14.5%) compared with luminal tumors (4.6%) was consistent with international data, which report prevalences of approximately 10–15% and about 5%, respectively [11].

This study has several strengths. First, it enrolled many patients from 46 institutions across Japan, ensuring sufficient sample size to produce highly precise and reliable results. Second, as a preregistration step, principal investigators at each site identified all surgical cases from 2023 that met the inclusion criteria, a process that likely minimized missed eligible cases.

However, we had several limitations. First, the participating facilities may not fully represent nationwide practice, limiting generalizability. All facilities had at least one breast cancer specialist, and more than half had genetic counselors, suggesting that the cohort was enriched for centers with well-established genetics-related infrastructure and expertise. Therefore, the testing rate observed in this study may overestimate the true uptake of *gBRCA* testing in routine clinical practice across Japan. Second, among patients with *gBRCA* PVs, a proportion did not receive adjuvant olaparib despite being identified through testing; however, detailed reasons for treatment decisions were not systematically collected in this study. Therefore, factors such as comorbidities, patient preference, toxicity concerns, or physician judgment could not be assessed and should be explored in future research. Third, some information was derived from medical records, raising the possibility of missing data or recall bias. Finally, we did not collect the reasons for non-testing directly from patients or their families, for example, through a questionnaire survey. Therefore, the patient-reported reasons for declining testing—for example, psychological factors or financial concerns—may not have been fully captured.

We propose three strategies to increase the uptake of *gBRCA* testing: (1) ensuring that HBOC diagnostic testing opportunities are not overlooked; (2) establishing a system that prevents eligible patients from being missed; and (3) providing repeated explanations from healthcare providers to patients at different time points.

The proportions of testing for HBOC diagnosis and for companion diagnostic purposes were comparable (Table S6), suggesting substantial overlap between the populations eligible for diagnostic and companion testing. Among 132 patients who underwent *gBRCA* testing despite not meeting the Japanese public insurance criteria for HBOC diagnosis, only 3 patients (2.4%) were found to carry *gBRCA* PVs. Thus, institutions with a well-integrated HBOC diagnostic pathway may be more likely to implement companion diagnostic testing consistently. Ideally, an electronic medical record (EMR) would automatically flag patients with high-risk, HER2-negative early breast cancer who are eligible for adjuvant olaparib. However, the feasibility of such automation remains limited, particularly in settings where EMR systems are not standardized across institutions—a challenge faced not only in Japan but in many healthcare systems worldwide. Therefore, multidisciplinary case review and educational efforts involving physicians, nurses, pharmacists, and patients may help improve the identification of eligible patients; however, their independent impact was not confirmed in the present study, and these should be regarded as hypothesis-generating implementation strategies that warrant further investigation in future studies.

Although *gBRCA* testing was performed in 98% of patients aged ≤ 40 years, the overall testing rate was only about 63%. The most common reason for non-testing was physician oversight, particularly in patients without features suggestive of HBOC. These findings highlight the need for greater physician awareness of companion diagnostics to ensure timely *gBRCA* testing and appropriate use of adjuvant olaparib.

## Supplementary Information

Below is the link to the electronic supplementary material.

Supplementary Material 1

Supplementary Material 2

## Declarations

### Generative AI and AI-assisted technologies in the manuscript preparation process

During the preparation of this work, the authors used ChatGPT (OpenAI, USA) in order to improve language and readability (English language editing and proofreading). After using this tool, the authors reviewed and edited the content as needed and take full responsibility for the content of the published article.
