# Supplementary material for: Real-world uptake of gBRCA testing as a companion diagnostic for olaparib in patients with high-risk HER2-negative early breast cancer in Japan: a cross-sectional multicenter study (BRCAwareness)
Source: Breast Cancer. 2026 May 23;33(4):919–27. doi: 10.1007/s12282-026-01867-y (PMC13283144; doi:10.1007/s12282-026-01867-y)
Supplement: Supplementary file 2 — Supplementary Material 2 [file 12282_2026_1867_MOESM2_ESM.pptx]

## Slide 1
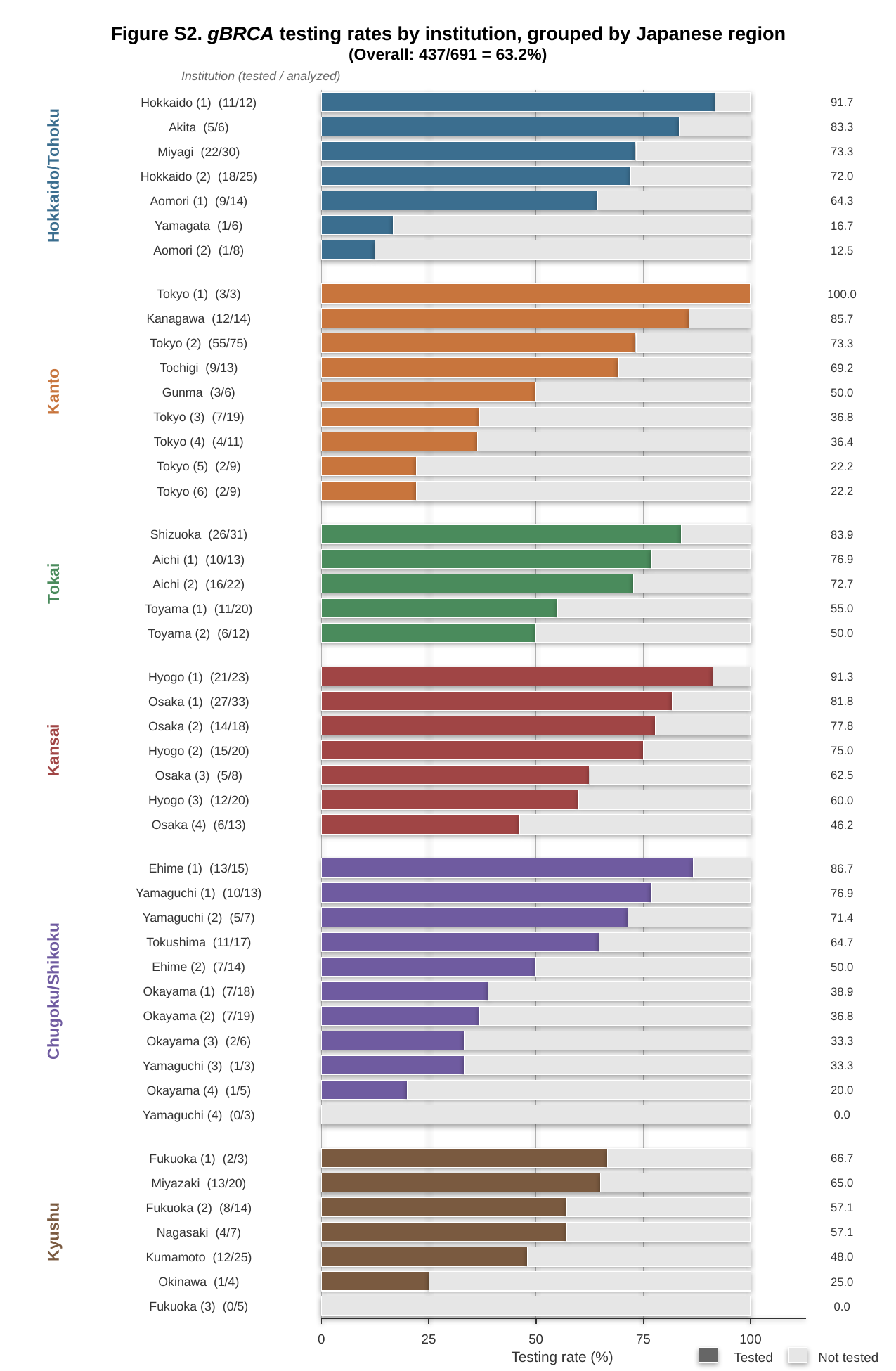

Figure S2. gBRCA testing rates by institution, grouped by Japanese region
(Overall: 437/691 = 63.2%)
Institution (tested / analyzed)
Hokkaido (1) (11/12)
91.7
Hokkaido/Tohoku
Akita (5/6)
83.3
Miyagi (22/30)
73.3
Hokkaido (2) (18/25)
72.0
Aomori (1) (9/14)
64.3
Yamagata (1/6)
16.7
Aomori (2) (1/8)
12.5
Tokyo (1) (3/3)
100.0
Kanto
Kanagawa (12/14)
85.7
Tokyo (2) (55/75)
73.3
Tochigi (9/13)
69.2
Gunma (3/6)
50.0
Tokyo (3) (7/19)
36.8
Tokyo (4) (4/11)
36.4
Tokyo (5) (2/9)
22.2
Tokyo (6) (2/9)
22.2
Shizuoka (26/31)
83.9
Tokai
Aichi (1) (10/13)
76.9
Aichi (2) (16/22)
72.7
Toyama (1) (11/20)
55.0
Toyama (2) (6/12)
50.0
Hyogo (1) (21/23)
91.3
Kansai
Osaka (1) (27/33)
81.8
Osaka (2) (14/18)
77.8
Hyogo (2) (15/20)
75.0
Osaka (3) (5/8)
62.5
Hyogo (3) (12/20)
60.0
Osaka (4) (6/13)
46.2
Ehime (1) (13/15)
86.7
Chugoku/Shikoku
Yamaguchi (1) (10/13)
76.9
Yamaguchi (2) (5/7)
71.4
Tokushima (11/17)
64.7
Ehime (2) (7/14)
50.0
Okayama (1) (7/18)
38.9
Okayama (2) (7/19)
36.8
Okayama (3) (2/6)
33.3
Yamaguchi (3) (1/3)
33.3
Okayama (4) (1/5)
20.0
Yamaguchi (4) (0/3)
0.0
Fukuoka (1) (2/3)
66.7
Kyushu
Miyazaki (13/20)
65.0
Fukuoka (2) (8/14)
57.1
Nagasaki (4/7)
57.1
Kumamoto (12/25)
48.0
Okinawa (1/4)
25.0
Fukuoka (3) (0/5)
0.0
0
25
50
75
100
Testing rate (%)
Tested
Not tested
